# Various Bee Pheromones Binding Affinity, Exclusive Chemosensillar Localization, and Key Amino Acid Sites Reveal the Distinctive Characteristics of Odorant-Binding Protein 11 in the Eastern Honey Bee, *Apis cerana*

**DOI:** 10.3389/fphys.2018.00422

**Published:** 2018-04-23

**Authors:** Xin-Mi Song, Lin-Ya Zhang, Xiao-Bin Fu, Fan Wu, Jing Tan, Hong-Liang Li

**Affiliations:** ^1^Zhejiang Provincial Key Laboratory of Biometrology and Inspection and Quarantine, College of Life Sciences, China Jiliang University, Hangzhou, China; ^2^College of Life Science, Shangrao Normal University, Shangrao, China

**Keywords:** *Apis cerana*, odorant-binding protein, transcriptional expression profile, immunofluorescence localization, fluorescence binding assay, site-directed mutagenesis

## Abstract

Odorant-binding proteins (OBPs) are the critical elements responsible for binding and transporting odors and pheromones in the sensitive olfactory system in insects. Honey bees are representative social insects that have complex odorants and pheromone communication systems relative to solitary insects. Here, we first cloned and characterized OBP11 (*AcerOBP11*), from the worker bees antennae of Eastern honey bee, *Apis cerana*. Based on sequence and phylogenetic analysis, most sequences homologous to AcerOBP11 belong to the typical OBPs family. The transcriptional expression profiles showed that AcerOBP11 was expressed throughout the developmental stages and highly specifically expressed in adult antennae. Using immunofluorescence localization, AcerOBP11 in worker bee's antennae was only localized in the sensilla basiconica (SB) near the fringe of each segment. Fluorescence ligand-binding assay showed that AcerOBP11 protein had strong binding affinity with the tested various bee pheromones components, including the main queen mandibular pheromones (QMPs), methyl p-hydroxybenzoate (HOB), and (*E*)-9-oxo-2-decanoic acid (9-ODA), alarm pheromone (n-hexanol), and worker pheromone components. AcerOBP11 also had strong binding affinity to plant volatiles, such as 4-Allylveratrole. Based on the docking and site-directed mutagenesis, two key amino acid residues (Ile97 and Ile140) were involved in the binding of AcerOBP11 to various bee pheromones. Taken together, we identified that AcerOBP11 was localized in a single type of antennal chemosensilla and had complex ligand-binding properties, which confer the dual-role with the primary characteristics of sensing various bee pheromones and secondary characteristics of sensing general odorants. This study not only prompts the theoretical basis of OBPs-mediated bee pheromones recognition of honey bee, but also extends the understanding of differences in pheromone communication between social and solitary insects.

## Introduction

Different with solitary insects, honey bees are typical social insects and bee colony generally has three types of bees (one queen, numerous workers, and several drones) (Plowes, 2010). As the core of the colony, the queen is a unique female bee that has the ability to breed offspring (after mating with drones) and assemble the whole of bee colony. The workers are in charge of rearing brood larvae, defending hives and foraging pollen and nectar (Pirk et al., 2011). Young adult worker bees always act as nurse bees for rearing brood larvae in the hive, and can change as foragers when older gradually (Weng et al., 2013). Bee colony always has quite complex pheromone cognitive system, which includes sex pheromones between virgin queen and drones, worker pheromones between worker bees, brood pheromones released from brood larvae, and alarm pheromones instantly released from guard worker bees when endangered (Pirk et al., 2011) and so on. Due to the hugeness of the numbers of bee colony, bee members have to utilize the bee pheromones to communicate each other in the hive. Therefore, bee pheromones and the corresponding sensing systems play a crucial role involved in regulating the complex social behavior of bee colonies.

In general, insects recognize odors or pheromones through their olfactory system. Odor molecules in the external environment are first carried by the odorant-binding proteins (OBPs) across the chemosensillar lymph, and then interact with the olfactory receptors (ORs) on the dendritic membrane of olfactory neurons, eventually resulting in electrical signals toward the central nervous system (Shanbhag et al., 1999; Brito et al., 2016). OBPs are low molecular weight, water-soluble globulins that transport odorant molecules across the lymph (Pelosi, 1996; Pelosi et al., 2017). In insects, OBPs can be divided into three subfamilies: pheromone-binding proteins (PBPs), general odorant-binding proteins (GOBPs), and antennal specific proteins (ASPs) or antennal-binding protein (ABPx) (Zhou, 2010).

Up to now, research on OBPs have focused on in solitary insects (Pelosi, 1996; Pelosi et al., 2017), such as Lepidoptera (Wang et al., 2004; Yang et al., 2016; Dong et al., 2017), Hemiptera (Sun et al., 2017; Wang et al., 2017), Blattodea (He et al., 2017), Diptera (Kim et al., 1998), and Coleoptera (Leal et al., 1998) etc. For typical social Hymenopteran, such as *Apis melliera*, its chemoreceptive system are obviously complex for its social behavior and life cycle. Based on the whole of *A. mellifera* genome (Honeybee Genome Sequencing Consortium, 2006), 21 OBPs were found and 9 *OBPs* of them were primarily expressed in antennae (Forêt and Maleszka, 2006). There are 171 olfactory receptors in the genome (Robertson and Wanner, 2006), and AmOr11 is the receptor for the major queen substance component 9-ODA (Wanner et al., 2007). As the functional studies, the OBP1 (ASP1) has been characterized as the queen pheromone-binding protein (Danty et al., 1999; Birlirakis et al., 2001; Pesenti et al., 2008). The OBP2 (ASP2) belong to GOBPs family (Danty et al., 1997; Briand et al., 2001), and OBP14 is a C-minus OBPs (having lost two conserved cysteines) (Zhou, 2010; Schwaighofer et al., 2014) etc. Recently, OBP11 in *A. mellifera*, was identified to be expressed in rare antennal sensilla basiconica in female bees, both workers and queens (Kucharski et al., 2016), while the physiological function of OBP11 associated with odor binding profiles is still unclear.

As the similar bee species of *A. mellifera, Apis cerana* is unique to China and capable of searching for sporadic nectar sources, and plays an important role in pollination of plants in mountainous areas (Radloff et al., 2010). Up to now, 17 OBPs have been found in *A. cerana* (Zhao et al., 2016). So far, three typical OBPs of them has been reported in-depth, ASP2 (OBP2) was specially distributed in worker bee antennae (Li et al., 2008), and bind the floral volatile with the dynamic binding mode (Li et al., 2013). ASP1 (OBP1) was expressed abundantly on the sensilla placoidea in drone antennae (Zhao et al., 2013b), and it can bind queen pheromone component with the static binding mode (Weng et al., 2015). *OBP11* have the highest expression in the stage of foragers, which display the highest olfactory sensitity in the *A. cerana* (Zhao et al., 2013a). In order to protect unique domestic bee resources in China, it is necessary to further study the physiological mechanisms of olfactory recognition system related to social behavior.

In this study, we successfully cloned *AcerOBP11* from the antennae of *A. cerana* worker bees. The expression profiles of AcerOBP11 in different developmental stages and tissues were determined by qRT-PCR, and the chemosensillar localization was observed in worker bee antennae. Moreover, we generated recombinant and mutant AcerOBP11 proteins, and identified that AcerOBP11 can bind to bee pheromones and related plant (floral) volatiles using a competitive fluorescence assay. We then predicted amino acids of AcerOBP11 that bind candidate ligands, and confirmed their role in ligand binding by molecular docking and site-directed mutagenesis. Our functional analysis of AcerOBP11 is of great significance to complement the characteristics of OBPs family of olfactory systems that are associated with *A. cerana's* unique social behavior.

## Mateials and methods

### Insects and tissue preparation

*A. cerana* colonies were maintained in Langstroth hives in Hangzhou city, Zhejiang province, China. The developmental stages of workers were classified following Michelette (Michelette and Soares, 1993). Antennae of 1,000 adult worker bees were pooled for RNA extraction of transcriptional sequencing. To analyze gene expression pattern during development, 100 worker eggs, 3 larvae, and 3 pupae were used for RNA extraction. To analyze gene expression in various adult tissues in different castes, antennae, head, thorax, abdomen, legs and wings from 1-day old workers, nurse workers (with feeding behavior of larvae, usually 6–18 days old) and forager workers (with carrying powder and pollination behavior, usually after 18 days of age) were used for RNA extraction, where each tissue/caste combination contained tissue from 50 worker bees.

### Plant volatiles and bee pheromones

All enzymes, kits and vectors, unless specified otherwise, were bought from TaKaRa (JP). ProteinIso® Ni-NTA Resin and fast mutagenesis system kit were purchased from Transgen Biotech Co. Ltd (Beijing, CN). Primers were synthesized from Sangon biotech Co. Ltd (Shanghai, CN), immunofluorescence related reagents were purchased from Beyotime Biotechnology (Shanghai, CN), plant volatiles and bee pheromones (purity > 97%) were purchased from J&K and TCI Technology Co., Ltd (Tokyo, JP). The rest of the reagents were domestic analytical reagents.

### Total RNA isolation, cDNA synthesis, and cloning of full-length AcerOBP11 cDNA

Total RNA was extracted from each tissue using Trizol (Invitrogen, US) according to the manufacturer's protocol. First-strand cDNA was synthesized using PrimeScript™ 1st Strand cDNA Synthesis Kit (TaKaRa, JP). Based on the *OBP11* homologous sequence of *A. mellifera* (GenBank accession: DQ435328.1), the full-length primer of AcerOBP11 was designed and *Bam*H I and *Xho* I restriction enzyme sites were introduced into the upstream and downstream primers. The upstream primer sequence was 5′-CCGGATCCATGAAAGCAGCAGAAAT-3′ and the downstream primer sequence was 5′-TTCTCGAGTCACGGAGCAATAAACGC-3′. The purified PCR products were subcloned into the pMD18-T vector (TaKaRa, JP) using a 1:3 (vector: PCR products) molar ratio by incubating the mixture with T4-DNA ligase at 4°C for 16 h. After transforming the ligation product into trans5α competent *E. coli* cells, the positive colonies were selected by white/blue screening and PCR with gene specific primers. Products were then submitted for sequencing company (Sangon, CN).

### Sequencing analysis and phylogenetic tree construction

The putative N-terminal signal peptides and cleavage site were predicted using SignalP V4.0 (http://www.cbs.dtu.dk/services/SignalP/) (Petersen et al., 2011). OBPs protein alignments were made using ClustalX V1.83 (Thompson et al., 1997) with default gap penalty parameters of gap opening 10 and extension 0.2, and were edited using ESPript (http://espript.ibcp.fr/ESPript/ESPript/) (Robert and Gouet, 2014). Phylogenetic tree was constructed by the neighbor joining method using MEGA V6.0 (http://www.megasoftware.net/) (Tamura et al., 2013) with bootstrap support of tree branches assessed by re-sampling amino acid positions 1,000 times.

### Quantitative real-time PCR (qRT-PCR)

Quantitative RT-PCR was performed using the iCycler iQ Real-Time PCR Detection System (Bio-Rad, US) with SYBR green dye (TaKaRa, JP). Experimental primers were qOBP11-F 5′-CTACGGAATACGGAGAA-3′ and qOBP11-R 5′-AATAAACGCTATGGGAT-3′, and control primers to amplify β*-Actin* was Be-Actin-F 5′-TCCTGCTATGTATGTCGC-3′ and Be-Actin-R was 5′-AGTTGCCATTTCCTGTTC-3′. The relative gene expression data were analyzed using the 2^−ΔΔ*CT*^ method by Livak (Livak and Schmittgen, 2001). Statistical analysis data (mean ± SE) from various samples (The developmental and tissues stage were analyzed, respectively) were subjected to one-way analysis of variance (ANOVA) followed by a least significant difference (LSD) test for mean comparisons. The significant differences were determined by *p*-values. Each experiment was performed in triplicates.

### Expression, purification, and confirmation of recombinant AcerOBP11 protein

AcerOBP11 was subcloned into the pET-32a (+) prokaryotic expression vector, and expressed in *E. coli* at high yields (>16 mg/L) through inducing by IPTG (the final concentration is 1 mmol/L). The AcerOBP11 recombinant protein was first expressed in the supernatant, then the denatured protein was purified using Ni^2+^ affinity chromatography for two rounds. After purification, the N-terminal tag was removed by enterokinase. The digested protein products were dialyzed 6–7 times with urea-free PBS dialysate (pH = 7.4) to obtain stable proteins with high purity. All purified AcerOBP11 recombinant protein was detected using standard SDS-PAGE method. The gel band containing the aim proteins was first cut out, digested by trypsin, and the detailed peptide sequences of the target proteins were identified using an LC-MS/MS mass spectrometry (Easy-nLC 1000 LTQ Obitrap ETD, Thermo Fisher, US). The secondary structure of purified AcerOBP11 recombinant protein was analyzed using circular dichroism (CD) spectrometry (815 type, Jasco, JP). Bradford assay was used to determine the concentration of AcerOBP11 and protein samples were stored in −20°C to generate polyclonal antisera and conduct the binding assays.

### Scanning electron microscopy (SEM)

For scanning electron microscopy (SEM), antennae of *A. cerana* worker bees were cleaned in 0.01 mol/L PBS (pH = 7.4) 3 times for 1 h. After treatment with 70% ethanol for 30 min, the samples were air-dried. The preparations were mounted on holders and examined using a SEM of XL30-ESEM (Philip, NL) after gold coating using a K500X sputter coater (Emitech, UK). Different sensilla types were classified following previously published criteria (Dietz and Humphreys, 1971).

### Fluorescence immunocytochemical localization

Female Bal B/C mice were repeatedly injected with AcerOBP11 recombinant protein emulsified in Freund's adjuvant, and antisera were obtained after 6–8 weeks and used without further purification. *A. cerana* worker foragers were collected from the hive, and their antennae were cut and embedded in OCT-Freeze medium. Antenna samples were sectioned using the Lecia-CM 1900 freezing microtome (Leica, DE). For the fluorescence immunocytochemical analysis, antennal sections were incubated with blocking buffer [1% BSA in TBS (contain 20% Tween-20, v/v)] for 1 h at RT, and then incubated with anti-*Acer*OBP11 antibodies in TBST at a 1:500 dilution for 1 h. After three washes with TBST, the sections were incubated with goat anti-mouse IgG conjugated with DyLight549 red dye (Beyotime, CN) at a 1:1,000 dilution in TBST for 1 h. The secondary antibodies of AcerOBP11 were used in the experiment as the negative control. After three washes with TBST, the sections were mounted in antifade mounting medium (Beyotime, CN) and observed with an Axio Observer Z1 microscope equipped with a LSM710 confocal laser scanning microscope (CarlZeiss, DE).

### Fluorescence competitive binding experiments

Fluorescence experiments on AcerOBP11 with N-phenyl-1-naphthylamine (1-NPN) were carried out on a Shimadzu RF-5301 spectrofluorimeter using a quartz cuvette in a right-angle configuration. The interactions were monitored by recording 1-NPN fluorescence upon addition of 1-NPN aliquots with excitation wavelength of 337 nm, and emission wavelength of 350–450 nm, where the slit was 5 nm. Titrations were carried out at 25°C with 1 μmol/L recombinant protein in PBS buffer. The fluorescence intensities at the maximum of emission wavelength (400~410 nm) were recorded to calculate Scatchard plots. The dissociation constant of the protein/1-NPN complex (*K*_1−NPN_) was calculated from Scatchard plots and applied in the equation below to estimate ligand binding affinity. All 23 ligands (1 mM) used in competition experiments were dissolved in spectrally pure grade methanol. Three independent measurements were taken for binding data. The concentrations of competitors that resulted in a reduction of fluorescence to half-maximal intensity (IC_*50*_ values), were taken as a measure of binding affinity constants calculated from the corresponding IC_50_ values using the following formula (Ban et al., 2003): *K*_D_ = [IC_*50*_]/(1+[1 – NPN]/*K*_1−NPN_), where [1-NPN] is the free concentration of 1-NPN and *K*_1−NPN_ is the dissociation constant of the complex AcerOBP11/1-NPN, which were calculated from the binding curve using the Origin 8.5 (OriginLab Inc.).

### Molecular docking

A 3D structure (Kiefer et al., 2009) of AcerOBP11 was predicted from *A. mellifera* odor binding protein 5 (AmOBP5) crystal structure (PDB entry code 3r72.1) using SWISS-MODEL online (https://www.swissmodel.expasy.org/). The 3D structures of all candidate pheromones and plant volatiles were obtained from NCBI PubChem online (https://pubchem.ncbi.nlm.nih.gov/). The 3D structure of the strongest binding ligand was docked with the predicted crystal structure of OBP11 via the Molegro Virtual Docker (MVD) 4.2 (free trial). The MolDock Optimizer and MolDock Score was used as the search criteria and grading standards, respectively (René and Christensen, 2006). The best docking model was selected for the pose display of OBP11 binding with candidate ligand. Residue distribution and hydrogen bond around AcerOBP11 were obtained when bound with a ligand to the key amino acid sites. Docking models were visualized with the UCSF Chimera package (Pettersen et al., 2004). Based on the docking analysis, the detailed energy values and hydrogen bonds involved in the binding of AcerOBP11 with ligands were calculated, and then displayed as a heat-map. The energy intensity was indicated as the depth of color, and the predicted hydrogen bonds were labeled using black frames.

### Site-directed mutagenesis and confirmation of key sites

In order to verify whether the predicted interaction sites played a role in the binding of AcerOBP11 protein with ligands, site-directed mutagenesis of the corresponding amino acid was carried out. The mutant primers were designed by a partial overlap method before synthesis. The plasmids of pET-32a/AcerOBP11 wild-type were mutated by using fast mutagenesis system kit (Transgen, CN) and then transformed into BL21 (*DE3*) competent cells. The mutant plasmids of target sites were confirmed by sequencing, and then the mutant proteins were obtained by the induction and purification in the same method as AcerOBP11 wild-type above. The secondary structure of purified AcerOBP11 recombinant protein mutants was also analyzed using circular dichroism (CD) spectrometry (815 type, Jasco, JP). The mutant proteins were used for competitive fluorescence experiments with the six candidate ligands chosen from the previous section. The target amino acids and binding mode of AcerOBP11 binding with candidate ligands were acquired by comparing the dissociation constant *K*_*D*_ between wild-type and mutant AcerOBP11. Statistical analysis data (mean ± SE) of *K*_*D*_ values for the same mutated amino acid site were also used as one-way analysis of variance (ANOVA) followed by a mean LSD test.

## Results

### Coding and amino acid sequences of AcerOBP11

**Figure 1:**
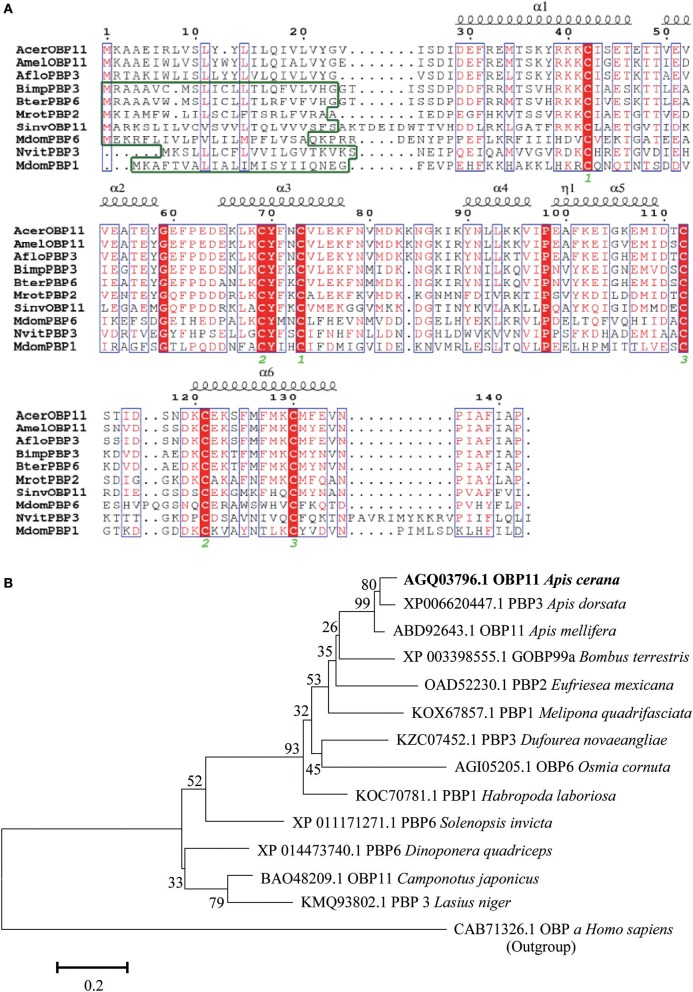
**(A)** Multiple amino acid sequences alignment of AcerOBP11 with significant homologous amino acid sequences from other species. Green box represents the amino acids sequences that do not contain a signal peptide. Red box represents conserved amino acids domains including six highly cysteines (labeled by green numbers below). The predicted secondary structures (e.g., α-helix) are shown above the corresponding sequences. **(B)** The phylogenetic tree of AcerOBP11 with other homologous proteins based on the method of Neighbor-Joining (Bootstrap = 1,000 times) using MEGA 6.0 software.

We cloned the coding sequence of *AcerOBP11* from *A. cerena*. The peptide did not contain predicted signal peptides by SignalP 4.1 Server (http://www.cbs.dtu.dk/services/SignalP/). We aligned the protein sequence with other homologous sequences and its predicted secondary structure, and found that AcerOBP11 contained amino acid sequence characteristics of OBPs, such as three pairs of disulfide bonds composed of six conserved Cysteines (Figure 1A). The *AcerOBP11* full-length ORF is 429 bp and the protein's molecular weight is approximately 15 kDa with an isoelectric point of 5.21, the GenBank accession of *AcerOBP11* was obtained as KC818631.1. The phylogenic tree (Figure 1B) showed that AcerOBP11 shared relative with some homologous OBPs from diverse Hymenopteran species. The amino acid sequence of AcerOBP11 shared highly similar to other PBPs, such as *A. mellifera* OBP11 (91%), *Apis dorsata* PBP3 (90%), *Eufriesea mexicana* PBP2 (79%), *Melipona quadrifasciata* PBP1 (66%), and *Bombus terrestris* GOBP 99a (72%) etc. It suggests that AcerOBP11 belongs to OBPs family in *A. cerana*, and has partly sequence characteristics of insect's PBPs and GOBPs.

### Transcriptional profiling of *AcerOBP11* in various tissues

**Figure 2:**
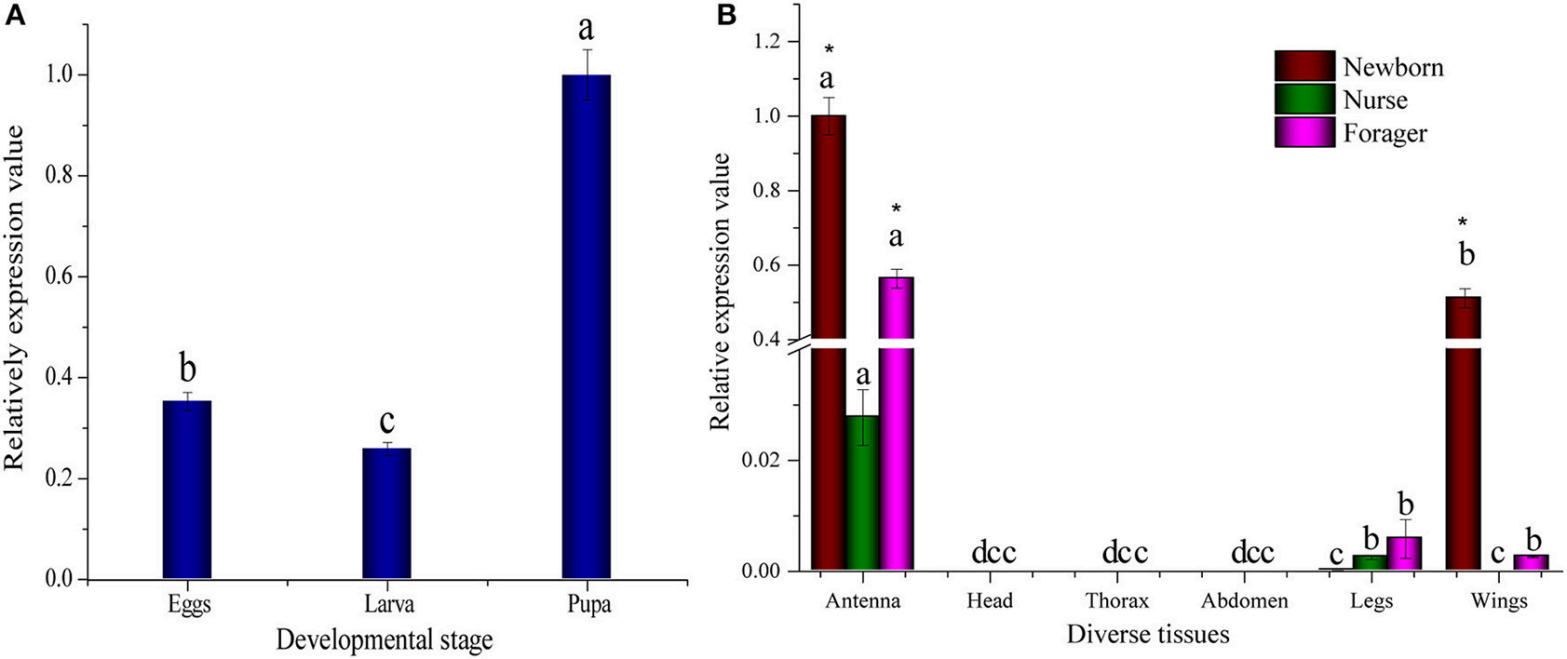
**(A)** Relative expression of *AcerOBP11* in different developmental stages. The graph represents mean ± *SD* normalized to the expression levels of *AcerOBP11* at pupal stage. **(B)** The relative expression of AcerOBP11 in different adult tissues, where graph represents mean ± *SD* normalized to the expression level in antennae of newborn worker. Newborn worker (color wine) marks 1-day old workers; the nurse (color olive) marks 6–18 days old workers; forager (color magenta) marks after 18 days old workers. ß*-actin* gene was used as reference to normalize the expression. The significant differences of the same tissue in different old workers are marked with different letters a, b, and c (*p* < 0.01, ANOVA). An asterisk indicates a significant difference of the diverse tissues in same old workers expression levels *(P* < 0.01, ANOVA).

We characterized the expression profiles of *AcerOBP11* in different tissues and developmental stages of *A. cerena* using real-time PCR. During the stage of development, *AcerOBP11* expression was higher in pupae than larvae and eggs (*p* < 0.01, ANOVA LSD; Figure 2A). In the adult workers, *AcerOBP11* was highly expressed in the antennae of newborn, nurse and forager bees (*p* < 0.01, ANOVA LSD). In addition, *AcerOBP11* showed high expression in the wing of newborn bees, low expression in legs of all three adult stages and low expression in the wings of the forager (Figure 2B). We found highest expression levels in the antennae, suggesting that *AcerOBP11* expression may be important in antennal physiological activity of worker bees. For different adult stages, we observed highest expression of *AcerOBP11* in newborn worker bees, followed by forager bees and nurse bees, indicating that *AcerOBP11* expression is dynamic in the antennae of adult worker bees.

### Preparation and confirmation of recombinant AcerOBP11 protein

**Figure 3:**
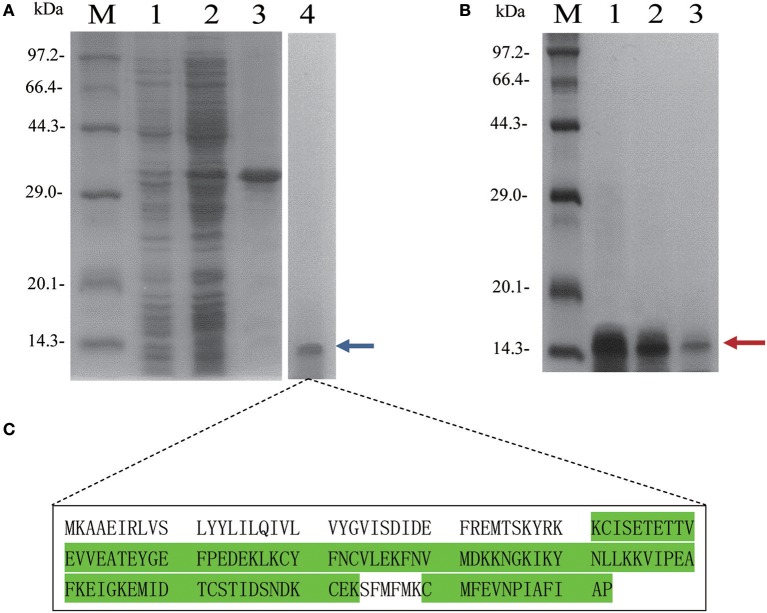
**(A)** Expression and purification of AcerOBP11 wild type proteins in *E. coli*. M is the protein molecular weight marker. Lane 1 and 2 represent whole lysate including pET32a-AcerOBP11 plasmid without and with induction of 1 mmol·L^−1^ IPTG, respectively. Lane 3 and 4 represent purified recombinant AcerOBP11 proteins before and after digestion with enterokinase, respectively. **(B)** The N-terminal tag of the mutant recombinant proteins are removed by enterokinase. M is the protein molecular weight marker. Lane 1–3 contains AcerOBP11m-Ile140, m-Phe101, and m-Ile97 mutant proteins after enterokinase digestion. The two rounds purified AcerOBP11 protein is labeled by red and blue arrow on the right, respectively. **(C)** The purified AcerOBP11 recombinant protein was identified by LC-MS/MS, and the green letters represent those amino acid sequences that have a total coverage of 67.17% with AcerOBP11 protein.

For the preparation of antibodies and the functional characterization of AcerOBP11, we induced and expressed *AcerOBP11* in *E. coli* and purified recombinant AcerOBP11 (Figure 3). The recombinant AcerOBP11 proteins without His-Tag were then purified through Ni^2+^ affinity chromatography column to obtain for subsequent experiments (Figure 3A, lane 4). The peptide sequence of the AcerOBP11 protein was identified using LC/MS-MS. As shown in Figure 3C, Figure S1, the identified peptide containing 37 peptides with high scores only belonged to the same AcerOBP11 protein group with a total coverage of 67.17%. It indicated that the purified AcerOBP11 recombinant protein should be integrated and errorless. Purified proteins were then used to generate mouse antibodies against AcerOBP11 and the fluorescence binding assay.

### Immunocytochemical localization

**Figure 4:**
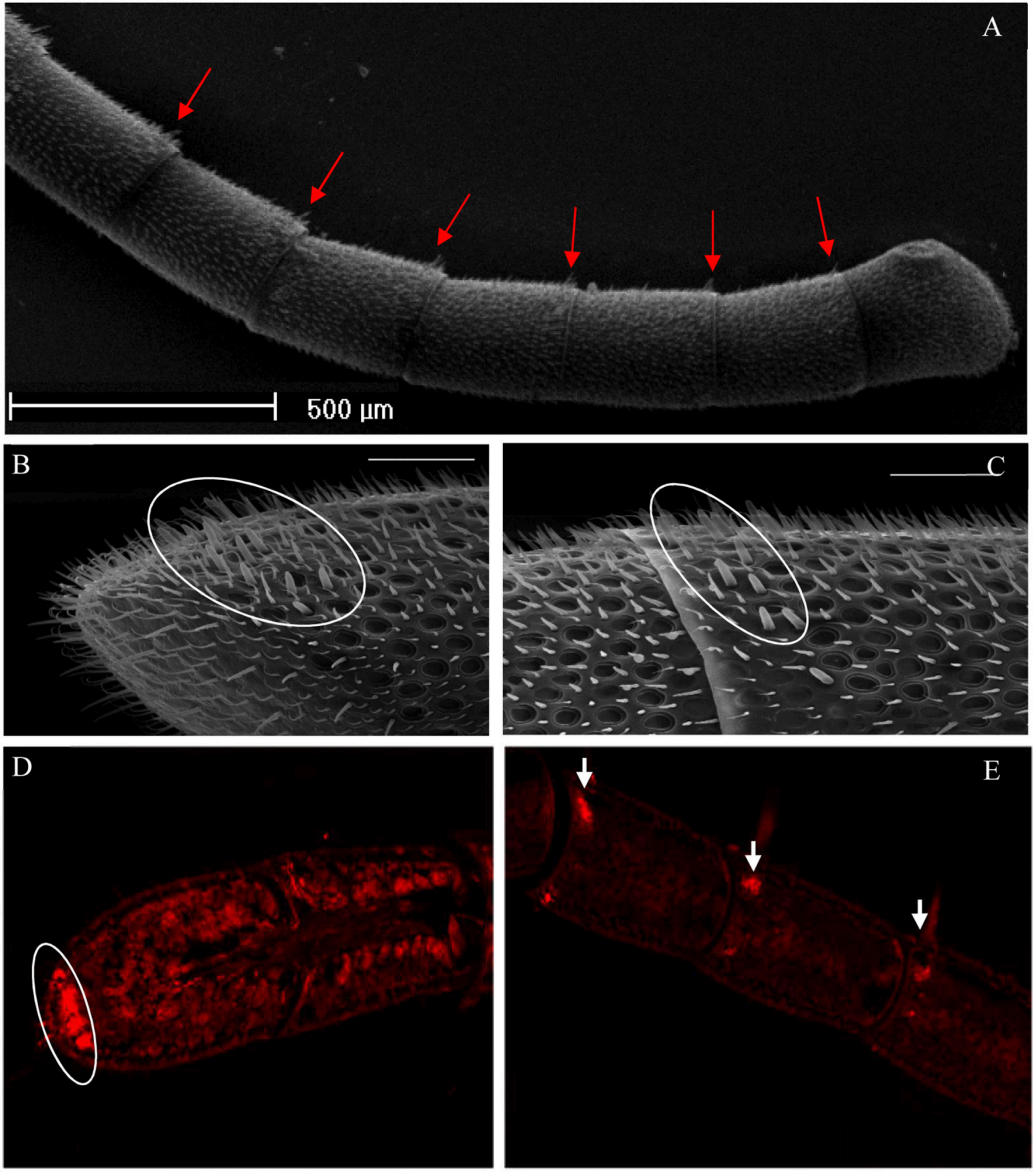
Immunofluorescence localization of AcerOBP11 in the forager worker antenna of *A. cerana*. **(A)** General antenna morphology, Scale bar = 500 μm. **(B)** First sub-segment of the antennae. **(C)** Third sub-segment of the middle of the antennae, where circle marks the *sensilla basiconica*. **(D,E)** White circles and arrows mark high localization of the protein, visible AcerOBP11 in the middle of the antennae only a specific distribution and the connection of each flagellum. **(B–E)** scale bar = 50 μm.

OBP proteins are generally expressed in the chemosensilla, and we found that *A. cerana* chemosensilla are primarily distributed on the antennal flagellum by SEM (Figure 4A). Using the newly generated AcerOBP11 antibodies, we conducted fluorescent-labeled immunocytochemical staining of the *A. cerana* worker bee antennae, and found high expression in the sensilla basiconica, but not in the sensilla trichoid and sensilla placodea on the antennae. AcerOBP11 expressing sensilla basiconica were mainly localized to the tip of antennae (Figures 4B,D), as well as restricted areas close to the interval between two segments on the antennal flagellum (Figures 4C,E). These results suggest that AcerOBP11 is specifically expressed in the antennal sensilla basiconica near the fringe of each segment in *A. cerana* worker bee.

### Ligand-binding assay of AcerOBP11

**Figure 5:**
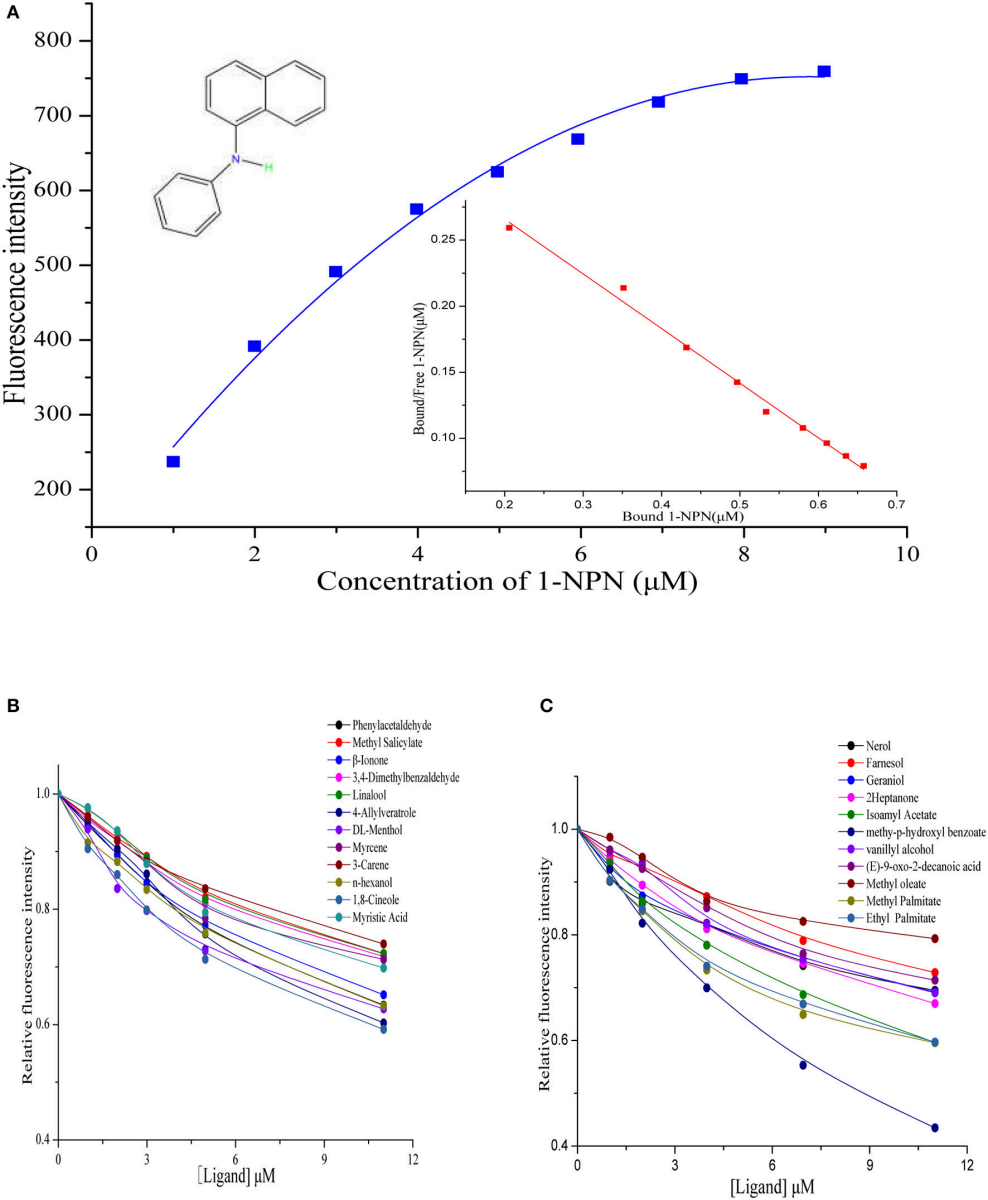
Binding affinity assay of AcerOBP11 to pheromone and plant volatiles. **(A)** The fluorescence intensity of AcerOBP11 with different concentrations of 1-NPN. **(B)** Competitive combination of plant volatiles with AcerOBP11. **(C)** Competitive combination of pheromones with AcerOBP11. Concentrations of all ligands are between 1 and 11 μmol/L.

Using the 1-NPN fluorescence reporter, we tested the binding affinity of AcerOBP11 to candidate plant volatiles and bee pheromones (Figure 5A, Table 1). The fluorescence competitor assay curve for each compound is shown in Figures 5B,C. All the values of dissociation constants were calculated and listed in Table 1. Among the 23 ligands in the assay, 22 candidate ligands except for methyl oleate reduced the relative fluorescence of 1-NPN to below 50% of AcerOBP11, indicating that AcerOBP11 bound to these compounds. The *K*_*D*_ values of QMP component (HOB), plant volatiles (4-hydroxyveratrole), and alarm pheromone (n-hexanol) were 1.35, 2.67, and 2.79 μmol/L, respectively. The three compounds had lower *K*_*D*_ values (<3 μmol/L), suggesting that they have stronger affinity to bind AcerOBP11. Moreover, the other bee alarm pheromones (isoamyl acetate), worker pheromone (farnesol), and brood pheromone (ethyl palmitate) were the strongest competitive ligands for 1-NPN in each group of components (Table 1). This is the first study about the function of OBPs in *A. cerana*.

**Table 1:** Fluorescence competitive assay of candidate ligands binding with recombinant AcerOBP11.

		**Ligands**	**The chemical structures**	**[I*C_*50*_*] (μM)**	***K_*D*_* (μM)**
Pheromones	Queen pheromone	HOB	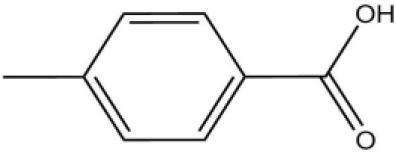	8.33	1.35
		9-ODA	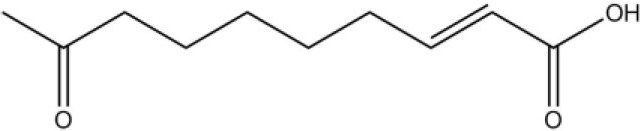	36.9	6.67
		HVA	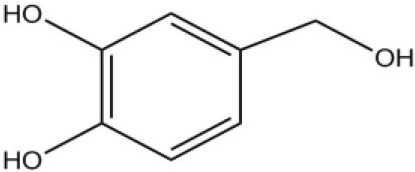	28.5	4.85
	Alarm pheromone	2-Heptanone	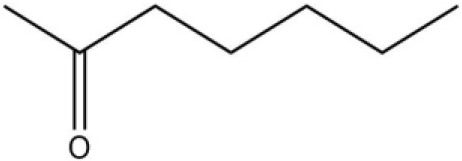	31.13	4.32
		Isoamyl acetate	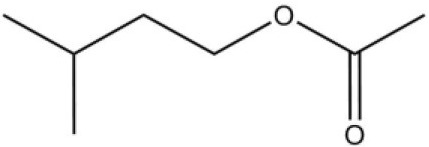	24.0	3.30
		n-hexanol	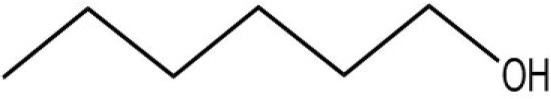	9.50	2.79
	Worker pheromone	Nerol	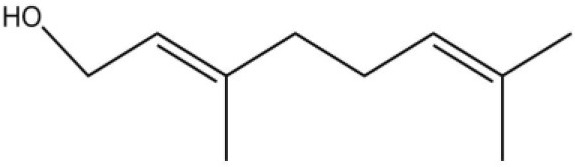	33.9	6.13
		Farnesol	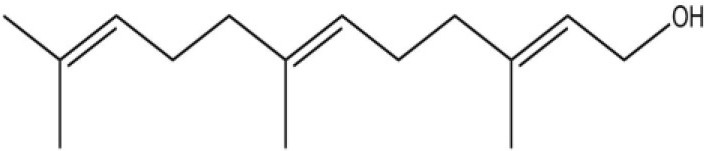	32.2	4.71
		Geraniol	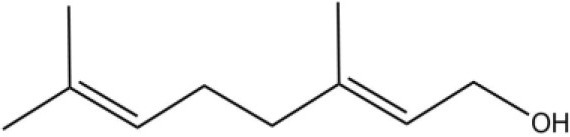	39.95	8.58
	Brood pheromone	Methyl palmitate	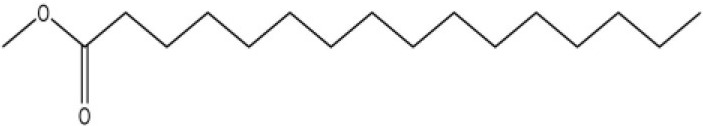	21	4.38
		Ethyl palmitate	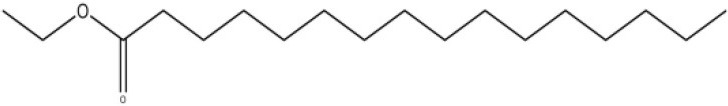	19.4	3.82
		Methyl oleate	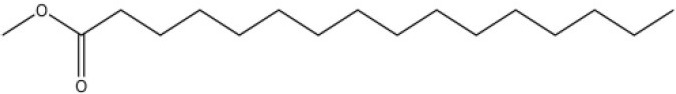	—	—
Plant volatiles		Phenylacetaldehyde	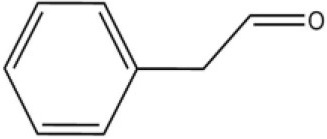	21.6	3.75
		Methyl salicylate	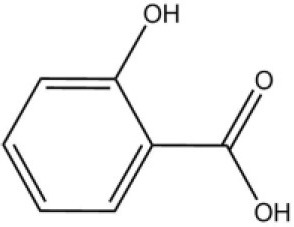	40	6.83
		β- Ionone	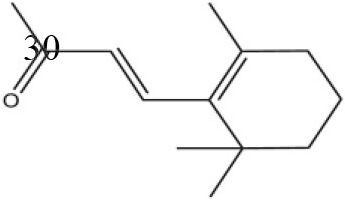	21.14	3.79
		3,4-Dimethylbenzaldehyde	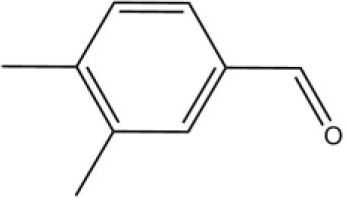	36.7	4.76
		Linalool	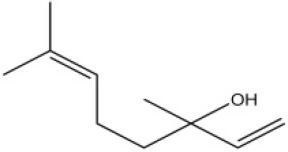	55	9.53
		4-Allylveratrole	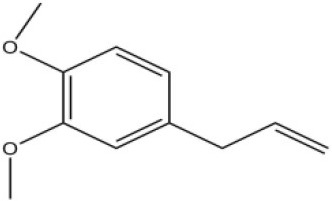	16.84	2.67
		DL-Menthol	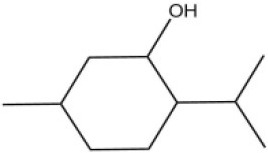	26.2	4.34
		Myrcene	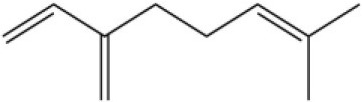	45.67	10.74
		3-Carene	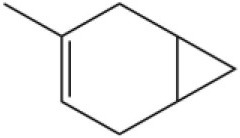	11.5	9.79
		1, 8-Cineole	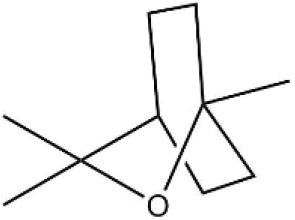	21.11	4.30
		Myristic acid	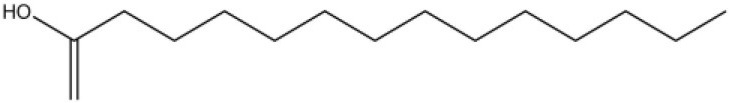	45.2	7.71

### Predicting key sites through analysis of docking and energy

**Figure 6:**
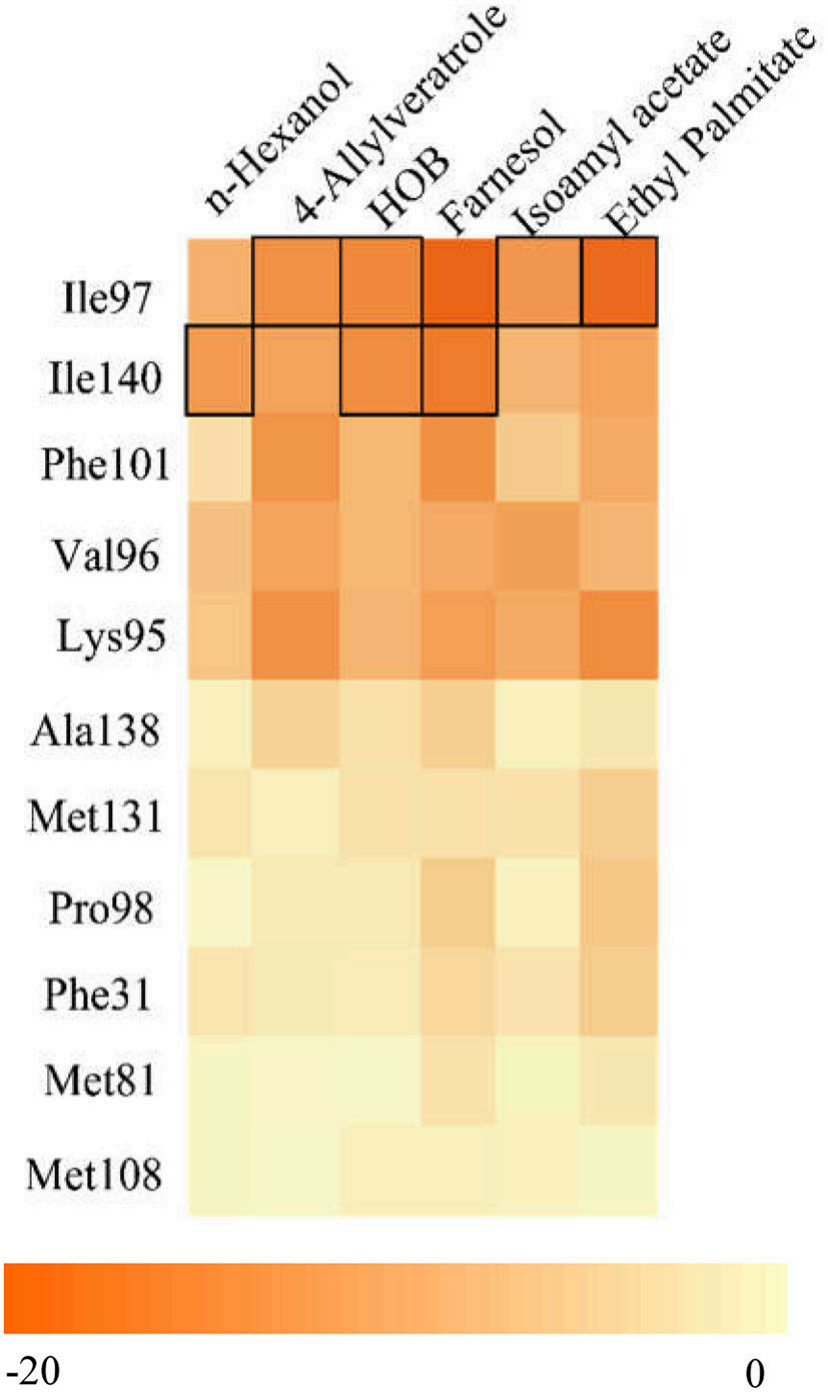
Heatmap of amino acid energy contribution in AcerOBP11 binding with six target ligands. The black boxes represent hydrogen bonds. Darker color indicates the larger contribution of the amino acid residue binding to the corresponding ligand.

Molecular docking can predict the interaction between proteins and small molecules. We generated a heat-map with detailed energy and hydrogen bond of the amino acids, and identified three amino acids, Ile97, Ile140, and Phe101, that are likely to play important roles in the binding of the AcerOBP11 to six ligands that had high affinity to AcerOBP11 (Figure 6, Table S1). In particular, Ile97 contributed a hydrogen bond for AcerOBP11 to bind to 4-hydroxyveratrole, HOB, isoamyl acetate and ethyl palmitate. Ile140 contributed a hydrogen bond for AcerOBP11 to bind to n-hexanol, HOB, and farnesol. Considering the energy contributions, we predicted that Ile97, Ile140, and Phe101 might be key amino acids in AcerOBP11 for its binding to ligands.

**Figure 7:**
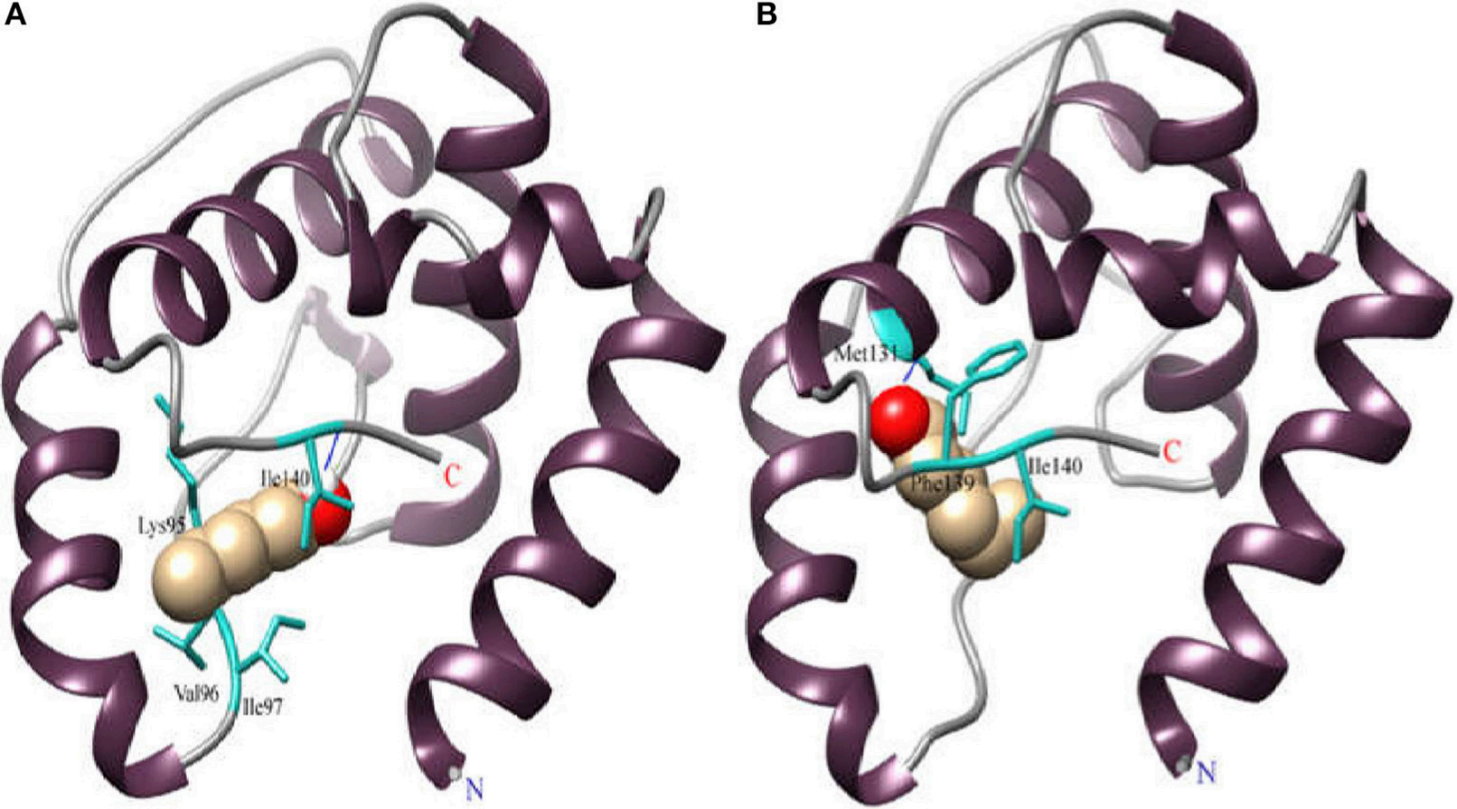
**(A)** Molecular docking of wild-type AcerOBP11 with n-hexanol. **(B)** Molecular docking of mutant AcerOBP11m-Ile97Gly with n-hexanol. The blue line represents the hydrogen bond. Cyan indicates predicted amino acids that play a role in hydrogen bond formation.

For the assessment of AcerOBP11 mutant with test ligand, a binding example of AcerOBP11 with n-hexanol was described. As displayed in Figure 7A, in AcerOBP11 wild-type, n-hexanol was located in a binding cavity composed of four hydrophobic amino acids of Ile97, Val96, Lys95, and Ile140. When Ile97 was mutated as glycine, the acting amino acids changed as the three hydrophobic amino acids, Ile140, Phe139, and Met131 close to the C-terminal (Figure 7B, Table S2). Compared with the AcerOBP11 wild-type, the hydrogen bond was changed from Ile140 to Met131. The number of key amino acids decreased and the location also changed. It indicates that Ile97 may play a key role in the binding of AcerOBP11 with n-hexanol.

### Confirming ligand binding sites through mutagenesis

**Figure 8:**
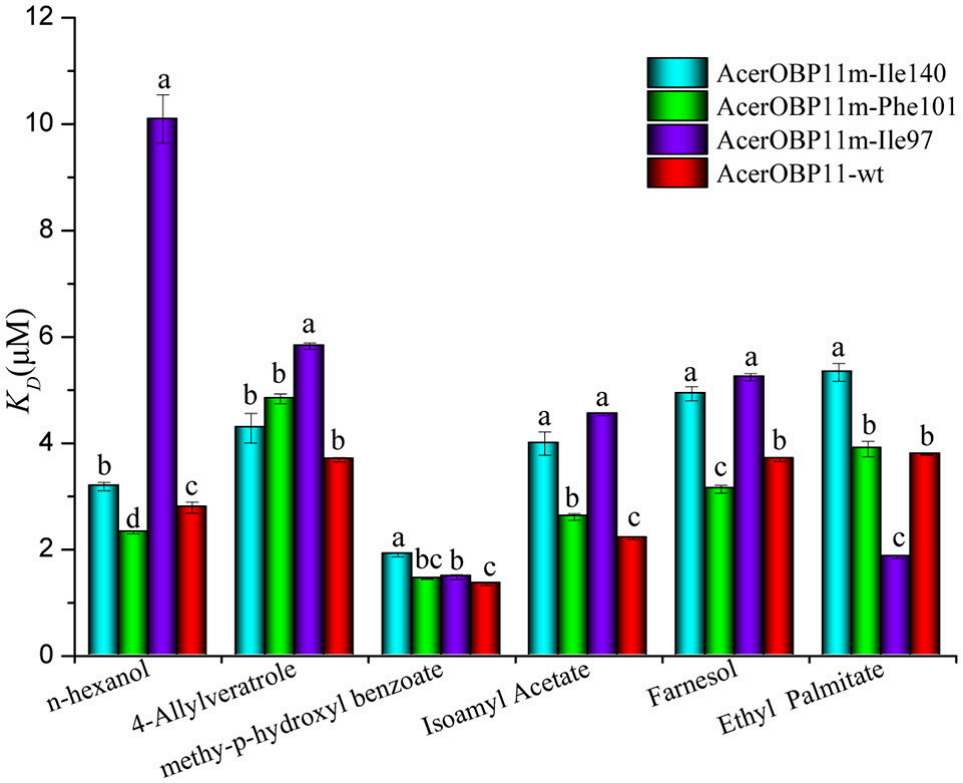
Dissociation of wild-type AcerOBP11 and Ile140Gly, Phe101Gly, and Ile97Gly mutant proteins to six ligands. Error bars represent the standard error. The significant differences of different proteins are marked with different letters a, b, c, and d (*p* < 0.01, ANOVA).

Using the fast mutagenesis system kit, we generated mutant AcerOBP11 by replacing amino acids Ile97, Ile140, and Phe101 with glycine (the corresponding primers listed in Table S3). All three AcerOBP11 mutant proteins were induced, purified, and confirmed by SDS-PAGE (Figure 3B, Figure S2). For the secondary structures, all three AcerOBP11 mutant proteins and wild-type showed the different degrees of protein characteristics by the confirmation of CD spectra (Figure S3). We performed competitive binding assays of the three mutant proteins with six candidate ligands to confirm the predicted role of these amino acids in binding to ligands. Compared with AcerOBP11-wt, the dissociation constant *K*_*D*_ of the AcerOBP11m-Ile97 mutation significantly increased for n-hexanol, 4-hydroxyveratrol, isoamyl acetate, and farnesol (Figure 8, the detailed data can be seen from Table S4). Especially with n-hexanol, the *K*_*D*_ of AcerOBP11m-Ile97 showed a significant 3.60-fold increase (*p* < 0.01, ANOVA). The *K*_*D*_ of AcerOBP11m-Ile140 mutant bound to the six chosen ligands also increased, and the largest increase of *K*_*D*_ was with isoamyl acetate (*K*_*D*_ increased 1.8-fold, *p* < 0.01, ANOVA, Figure 8). However, we did not observe significant increases in of AcerOBP11m-Phe101 when bound to 4-hydroxyveratrol, HOB, isoamyl acetate, and ethyl palmitate. In summary, Ile97 and Ile140 appear to be the key binding sites of AcerOBP11 with candidate ligands.

## Discussion

Social insects possess complex pheromone-driven behaviors that are regulated by chemical communication systems, regulating the social activities of the whole colony (Pankiw et al., 2004). Here, we cloned and functionally characterized a OBPs gene, *AcerOBP11*, from the antennae of *A. cerana*. It did not contain a signal peptide, and showed high similarity with homologous proteins in other insect OBPs (Figure 1). According to the sequence alignments and phylogenic tree analysis, it suggests that *AcerOBP11* belongs to a typical odorant-binding protein family in *A. cerana*.

In *A. mellifera*, OBP11 is highly expressed in the antennae of forager workers and queens, and is not expressed in the egg, larval, and pupal stages (Forêt and Maleszka, 2006). *AcerOBP11* showed high expression in pupae compared with eggs and larvae (Figure 2A, *p* < 0.01, *t*-test). Ligand-binding assay showed that AcerOBP11 could bind with some brood pheromone components (Table 1) that is released from larvae and sensed by nurse bees in the hive. It suggests that AcerOBP11 may be relevant to the synthesis and transportation of brood pheromones in these two stages. *AcerOBP11* was also abundantly expressed in the antennae and wings of newborn workers, suggesting that AcerOBP11 may play a role in newly eclosed *A. cerana*.

In particular, *AcerOBP11* was highly expressed in the antennae of worker bee at various ages (Figure 2B, *p* < 0.01, *t*-test), strongly indicating that it is involved in the olfactory behavior of worker bees. *AcerOBP11* expression was higher in forager bees than nurse bees (Figure 2B), and this may be related to the behavioral activity for foraging honey and pollen. This expression results was almost consistent with the previous reports (Zhao et al., 2013a, 2016). Therefore, AcerOBP11 may be involved in the eclosion of worker bee and olfactory sensing functions during the forager stage.

AmelOBP11 was distributed only in sensilla basiconica at the top of antenna 3–10 segments (Kucharski et al., 2016). Our results are consistent with this finding that AcerOBP11 was localized to the antennal sensilla basiconica near the top of each segment in *A. cerana* (Figures 4D,E). Based on the external morphology of antennal sensilla of Apoidea, sensillar basiconica in bee antennae is likely involved in olfactory functions (Galvani et al., 2012). In the same Hymenoptera social insect, *Camponotus japonicus*, sensilla basiconica can recognize cuticular hydrocarbon (CH) pheromones to determine nest-mates and non-nest-mates (Ozaki et al., 2005). Furthermore, the GOBPs protein ASP2 in *A. cerana* is mainly expressed in sensilla placodea and plays a typical olfactory role in sensing general odors (Li et al., 2008). Overall, considering that AcerOBP11 was specially expressed in the sensilla basiconica (rather than sensilla placodea), it is likely that AcerOBP11 tends to the primary characteristics of bee pheromones sensing and the secondary characteristics of insect GOBPs.

In all 23 candidate chemical pheromones and plant volatiles that were tested for AcerOBP11 binding in this study, we found that 12 bee pheromones bound to AcerOBP11 (except for methyl oleate). The queen mandibular pheromone (QMP) components HOB, 9-ODA, and HVA showed high affinity to AcerOBP11 with *K*_*D*_ values < 10 μmol/L. HOB showed highest binding affinity to AcerOBP11 (*K*_*D*_ = 1.35 μmol/L). 9-ODA is the typical bee sex pheromone that drones perceive, and 9-ODA is released by virgin queens to induce courtship and mating in males (Brockmann et al., 2006; Villar et al., 2015). HVA is a unique QMPs component in the western honey bee (Plettner et al., 1997), and also bound strongly to OBP11 here. The QMPs component can inhibit and regulate the ovary development of worker bees (Hoover et al., 2003; Peso et al., 2013), regulate programmed cell death in worker bee ovaries (Ronai et al., 2016), and affect activation of the worker bee ovary and ovarian duct (Ken et al., 2015). Therefore, the high affinity of AcerOBP11 with QMPs components suggests that AcerOBP11 may play an important role in the process of worker bees sensing the QMPs released by queen, and then affect the regulation of bee colony.

In addition, AcerOBP11 also bound strongly to brood pheromone components methyl palmitate and ethyl palmitate, rather than to another component methyl oleate (Table 1). Brood pheromones can inhibit ovarian development in worker bees (Arnold et al., 1994), stimulate and regulate pollen foraging activity of bees (Pankiw et al., 1998; Pankiw, 2004), and induce release of pheromones by the queen (Mohammedi et al., 1996). Moreover, AcerOBP11 strongly bound to alarm pheromones and worker pheromones (Table 1), suggesting that AcerOBP11 may play a role in worker bee behavior to maintain and defend the colony. Considering the high expression level of *AcerOBP11* in nurse and forager bees, and the high affinity of AcerOBP11 with a variety of bee pheromones suggests that AcerOBP11 is an odorant-binding protein that can sense and regulate bee pheromones that are important to the *A. cerana* colony.

In addition to sensing bee pheromones, in this study, we found that AcerOBP11 can strongly bind with 11 plant volatiles (Figure 5C, Table 1). For example, β-ionone is a volatile produced in flowering plants (Li et al., 2013), and had higher binding affinity with AcerOBP11 (*K*_*D*_ = 3.78 μmol/L) than ASP1 (*K*_*D*_ = 14.69 μmol/L) (Weng et al., 2013) and ASP2 (*K*_*D*_ = 5.14 μmol/L) (Li et al., 2013). This suggests that AcerOBP11 may be involved in olfactory orientation for searching nectar sources, consistent with the results that *AcerOBP11* is highly expressed in forager bee antennae (Figure 2). In conclusion, based on the integrated immunolocalization and functional studies of binding with bee pheromone and plant volatiles, AcerOBP11 was identified to play a dual-role that it had the primary characteristics of sensing various bee pheromones and secondary characteristics of sensing general odorants.

Molecular docking and site-directed mutagenesis can reveal amino acids that mediate ligand binding (Pelosi et al., 2014; Lu et al., 2015; Zhu et al., 2016). We identified Ile97, Ile140, and Phe101 are potential regulators of ligand binding, where Ile97 and Ile140 contributed hydrogen bonds to the ligand (Figure 6). Using AcerOBP11 mutant proteins, we found that binding affinity significantly decreased when Ile97 and Ile140 were mutated (Figure 8), suggesting that Ile97 and Ile140 may mediate AcerOBP11 binding with some ligands. In addition, we noticed that in the multiple sequence alignments the same positions of No. 97 and 140 of AcerOBP11 were always shown as Leu97/140 or Phe140 in other sequences, instead of Ile97/140 (Figure 1A). When we manually substituted Ile97/140 to Leu97/140 or Phe140, respectively, the Moldock scores and hydrogen bonds energies were shown by the analysis of docking (Figure S4). It was evidently that the energies of all AcerOBP11 wild-type were always the lowest, the mutants of m-Ile97/140Gly were the highest, whereas the other predicted mutants of m-Ile97Leu/Ile140Leu(Phe) had slightly higher energies than AcerOBP11 wild-type. It suggests that the alkane chain in isoleucines (Ile97/140) or leucines (Leu97/140) might play significant role in the interactions between AcerOBP11 and ligands.

Hydrogen bond is usually one of the most common forces that bind proteins with small molecules (Jiang et al., 2009; Zhuang et al., 2014; Li et al., 2016). In AcerOBP11, Ile140 contributes to the unique hydrogen with n-hexanol (Figures 6, 7), while the hydrophobic amino acid Ile97 plays a major role rather than Ile140 according to the results of mutagenesis (Figure 8). These results suggest that hydrophobic interactions between AcerOBP11 and its ligands are critical for binding, especially for interactions between AcerOBP11 and n-hexanol, similar to findings from ASP2 and imidacloprid in *A. cerana* (Li et al., 2015), implying that complex interactions take place between olfactory proteins and compounds involved in the cognitive system of social insects.

## Author contributions

X-MS and H-LL: conceived and designed the experiments; X-MS and L-YZ: performed the experiments; X-BF, JT, and FW: analyzed the data; X-MS and H-LL: wrote the manuscript.

### Conflict of interest statement

The authors declare that the research was conducted in the absence of any commercial or financial relationships that could be construed as a potential conflict of interest.
