# Supplementary material for: Various Bee Pheromones Binding Affinity, Exclusive Chemosensillar Localization, and Key Amino Acid Sites Reveal the Distinctive Characteristics of Odorant-Binding Protein 11 in the Eastern Honey Bee, Apis cerana
Source: Front Physiol. 2018 Apr 23;9:422. doi: 10.3389/fphys.2018.00422 (PMC5924804; doi:10.3389/fphys.2018.00422)
Supplement: Supplementary file 1 [file Table1.PDF]

Table S1. The predicted amino acid energy contribution of *Acer*OBP11 binding with ligands

	n-Hexanol	4-Allylveratrole	HOB	Farnesol	Isoamyl acetate	Ethyl palmitate
Ile97	-9.564	-13.991	-15.116	-20.489	-12.872	-19.501
Ile140	-12.649	-11.492	-14.322	-17.104	-9.076	-11.415
Phe101	-3.614	-13.637	-8.848	-14.083	-6.458	-10.615
Val96	-7.435	-11.503	-8.880	-10.847	-11.723	-8.963
Lys95	-7.015	-13.745	-9.057	-12.248	-10.402	-14.651
Ala138	-1.281	-5.274	-3.506	-5.665	-1.238	-2.672
Met131	-2.972	-1.571	-3.270	-3.552	-3.167	-5.788
Pro98	-0.502	-2.330	-2.380	-6.138	-1.048	-6.661
Phe31	-2.929	-2.249	-2.041	-4.710	-2.995	-6.207
Met81	-0.835	-0.595	-0.314	-3.165	-1.003	-2.587
Met108	-0.916	-0.350	-1.410	-1.411	-1.099	-0.738

Note: The amino acids are predicted to produce hydrogen bonds are marked with blue letters.
