# Supplementary material for: Various Bee Pheromones Binding Affinity, Exclusive Chemosensillar Localization, and Key Amino Acid Sites Reveal the Distinctive Characteristics of Odorant-Binding Protein 11 in the Eastern Honey Bee, Apis cerana
Source: Front Physiol. 2018 Apr 23;9:422. doi: 10.3389/fphys.2018.00422 (PMC5924804; doi:10.3389/fphys.2018.00422)
Supplement: Supplementary file 2 [file Table2.PDF]

Table S2. The amino acid energy contribution in the process of *Acer*OBP11-wt and *Acer*OBP11-Ile97 binding with n-Hexanol

| AcerOBP11-wt | | AcerOBP11-Ile97 | |
|---|---|---|---|
| Amino acid | Energy | Amino acid | Energy |
| Ile140 | -12.649 | Ile140 | -13.008 |
| Ile97 | -9.564 | Phe139 | -9.808 |
| Val96 | -7.435 | Met131 | -8.029 |
| Lys95 | -7.015 | Met108 | -3.638 |
| Phe101 | -3.614 | Ala138 | -3.315 |
| Met131 | -2.972 | Lys95 | -3.260 |
| Phe31 | -2.929 | Asn135 | -2.629 |
| Ala138 | -1.281 | Phe132 | -1.523 |

Note: The predicted key amino acids are marked with red letters.
