# Supplementary material for: Various Bee Pheromones Binding Affinity, Exclusive Chemosensillar Localization, and Key Amino Acid Sites Reveal the Distinctive Characteristics of Odorant-Binding Protein 11 in the Eastern Honey Bee, Apis cerana
Source: Front Physiol. 2018 Apr 23;9:422. doi: 10.3389/fphys.2018.00422 (PMC5924804; doi:10.3389/fphys.2018.00422)
Supplement: Supplementary file 3 [file Table3.PDF]

Table S3. The site-directed mutagenesis primers of *AcerOBP11*

Primers name	5'→3'
AcerOBP11-Ile140-F	**GTCAATCCTATAGCGTTT**ggTGCTCCGTG
AcerOBP11-Ile140-R	cc**AAACGCTATAGGATTGAC**TTCGAACATAC
AcerOBP11-Ile97-F	**TTACTGAAAAAAGTA**ggTCCAGAAGCA
AcerOBP11-Ile97-R	cc**TACTTTTTTCAGTAA**ATTATATTT
AcerOBP11-Phe101-F	**GTAATTCCAGAAGCA**ggTAAAGAAATAGG
AcerOBP11-Phe101-R	cc**TGCTTCTGGAATTAC**TTTTTTCAGTAA

Note: The nucleotide sites to be mutated are marked with cyan letters, and the overlapping complementary sequences between sense and anti-sense primers are marked with bold letters.
