# Supplementary material for: Various Bee Pheromones Binding Affinity, Exclusive Chemosensillar Localization, and Key Amino Acid Sites Reveal the Distinctive Characteristics of Odorant-Binding Protein 11 in the Eastern Honey Bee, Apis cerana
Source: Front Physiol. 2018 Apr 23;9:422. doi: 10.3389/fphys.2018.00422 (PMC5924804; doi:10.3389/fphys.2018.00422)
Supplement: Supplementary file 4 [file Table4.PDF]

Table S4. The K_D and $[IC_{50}]$ of competitively binding of six chosen ligands with 1-NPN and recombinant *Acer*OBP11 and the three site-directed mutagenesis proteins

	AcerOBP11-wt		AcerOBP11m-Ile140		AcerOBP11m-Phe101		AcerOBP11m-Ile97	
	[I*C50*] (μM)	K_D (μM)	[I*C50*] (μM)	K_D (μM)	[I*C50*] (μM)	K_D (μM)	[I*C50*] (μM)	K_D (μM)
n-Hexanol	21.117±0.785	2.793±0.104	11.813±0.283	3.186±0.076	14.257±0.124	2.321±0.020	33.400±0.785	10.096±0.456
HOB	8.313±0.156	1.35±0.022	5.567±0.114	1.906±0.039	6.257±0.025	1.447±0.006	6.090±0.156	1.483±0.045
Isoamyl acetate	16.877±0.144	2.212±0.019	12.870±0.697	3.995±0.221	10.740±0.26	2.615±0.063	17.750±0.144	4.544±0.022
Farnesol	20.553±0.237	3.698±0.042	19.895±0.536	4.931±0.133	17.783±0.411	3.138±0.072	21.340±0.237	5.241±0.069
Ethyl palmitate	24.393±0.076	3.788±0.012	16.637±0.520	5.335±0.167	20.280±0.747	3.893±0.143	6.610±0.076	1.860±0.022
4-Allylveratrole	21.017±0.22	3.605±0.038	22.493±1.463	4.282±0.279	20.087±0.381	4.836±0.092	25.823±0.022	5.826±0.060
