# Supplementary material for: Various Bee Pheromones Binding Affinity, Exclusive Chemosensillar Localization, and Key Amino Acid Sites Reveal the Distinctive Characteristics of Odorant-Binding Protein 11 in the Eastern Honey Bee, Apis cerana
Source: Front Physiol. 2018 Apr 23;9:422. doi: 10.3389/fphys.2018.00422 (PMC5924804; doi:10.3389/fphys.2018.00422)
Supplement: Supplementary file 5 [file Image1.pdf]

Figure S1. Peptide sequences of recombinant AcerOBP11 protein analyzed by LC-MS/MS.

Accession	Description	Score	Coverage	# Proteins	# Unique Peptides	# Peptides
sp	Apis cerana cerana	537.21	67.61	1	21	21
	A2	Sequence	# PSMs	# Proteins	# Protein Groups	Protein Group Accessions
	High	KcISETETTVEVVEATEYGEFPEDEKLK	2	1	1	sp
	High	KcISETETTVEVVEATEYGEFPEDEK	2	1	1	sp
	High	cISETETTVEVVEATEYGEFPEDEKLK	4	1	1	sp
	High	EIGKEmIDTcSTIDSNDKcEK	15	1	1	sp
	High	EIGKEmIDTcSTIDSnDKcEK	6	1	1	sp
	High	cISETETTVEVVEATEYGEFPEDEK	3	1	1	sp
	High	EMIDTcSTIDSnDKcEK	1	1	1	sp
	High	EMIDTcSTIDSNDKcEK	10	1	1	sp
	High	EmIDTcSTIDSNDKcEK	18	1	1	sp
	High	EIGKEmIDTcSTIDSNDK	4	1	1	sp
	High	EIGKEMIDTcSTIDSnDKcEK	3	1	1	sp
	High	cYFNcVLEKFNVmDK	1	1	1	sp
	High	YNLLKKVIPEAFK	1	1	1	sp
	High	EmIDTcSTIDSNDK	8	1	1	sp
	High	EmIDTcSTIDSnDKcEK	1	1	1	sp
	High	EIGKEMIDTcSTIDSNDK	2	1	1	sp
	High	KVIPEAFKEIGK	1	1	1	sp
	High	EIGKEMIDTcSTIDSNDKcEK	5	1	1	sp
	High	cYFNcVLEK	6	1	1	sp
	High	EIGKEMIDTcSTIDSnDK	1	1	1	sp
	High	FNVMDKKnGK	3	1	1	sp
	High	FNVmDKKnGK	2	1	1	sp
	High	VIPEAFKEIGK	2	1	1	sp
	High	EMIDTcSTIDSNDK	8	1	1	sp
	High	cYFncVLEK	2	1	1	sp
	High	KVIPEAFK	25	1	1	sp
	High	NGKIKYNLLK	1	1	1	sp
	High	IKYNLLKK	1	1	1	sp
	High	nGKIKYNLLK	2	1	1	sp
	High	FNVMDKKNGK	1	1	1	sp
	High	IKYNLLK	2	1	1	sp
	High	IKYnLLK	1	1	1	sp
	High	FNVMDKK	6	1	1	sp
	High	FNVmDKK	7	1	1	sp
	High	EMIDTcSTIDSnDK	1	1	1	sp
	High	YNLLKK	1	1	1	sp
	High	cMFEVNPIAFIAP	1	1	1	sp

# PSMs	# AAs	MW [kDa]	calc. pI
160	142	16.5	5.25

Modifications	ΔCn	XCorr	Charge	MH+ [Da]	ΔM [ppm]	RT [min]	# Missed Cleavages
C2(Carbamidomethyl)	0.0000	9.43	3	3260.55210	3.59	80.78	2
C2(Carbamidomethyl)	0.0000	7.56	3	3019.36264	0.42	81.36	1
C1(Carbamidomethyl)	0.0000	7.56	4	3132.47158	8.35	83.55	1
M6(Oxidation); C10(Carbamidomethyl); C19(Carbamidomethyl)	0.0000	7.39	3	2489.09488	4.31	37.71	2
M6(Oxidation); C10(Carbamidomethyl); N16(Deamidated); C19(Carbamidomethyl)	0.0000	7.32	3	2490.09500	10.78	38.25	2
C1(Carbamidomethyl)	0.0000	7.20	3	2891.28971	8.06	84.13	0
C6(Carbamidomethyl); N12(Deamidated); C15(Carbamidomethyl)	0.0000	6.65	3	2046.84464	7.08	43.05	1
C6(Carbamidomethyl); C15(Carbamidomethyl)	0.0000	6.36	3	2045.86301	8.25	41.13	1
M2(Oxidation); C6(Carbamidomethyl); C15(Carbamidomethyl)	0.0000	5.83	3	2061.85013	4.41	39.32	1
M6(Oxidation); C10(Carbamidomethyl)	0.0000	5.37	3	2071.93082	7.19	42.12	1
C10(Carbamidomethyl); N16(Deamidated); C19(Carbamidomethyl)	0.0000	5.00	3	2474.08188	3.49	55.47	2
C1(Carbamidomethyl); C5(Carbamidomethyl); M13(Oxidation)	0.0000	4.87	3	1982.89414	6.63	83.46	1
	0.0000	4.81	3	1562.94248	7.64	79.48	2
M2(Oxidation); C6(Carbamidomethyl)	0.0000	4.62	2	1644.67461	1.09	37.77	0
M2(Oxidation); C6(Carbamidomethyl); N12(Deamidated); C15(Carbamidomethyl)	0.0000	4.54	2	2062.84355	8.96	31.88	1
C10(Carbamidomethyl)	0.0000	4.34	3	2055.93125	4.98	58.83	1
	0.0000	4.13	3	1358.81256	6.09	57.83	2
C10(Carbamidomethyl); C19(Carbamidomethyl)	0.0000	4.04	3	2473.08927	0.01	50.19	2
C1(Carbamidomethyl); C5(Carbamidomethyl)	0.0000	3.60	2	1232.55120	5.94	73.03	0
C10(Carbamidomethyl); N16(Deamidated)	0.0000	3.59	3	2056.91654	5.59	60.86	1
N8(Deamidated)	0.0000	3.46	3	1181.60410	4.83	23.59	2
M4(Oxidation); N8(Deamidated)	0.0000	3.39	2	1197.59856	4.39	15.41	2
	0.0000	3.32	3	1230.71656	5.88	69.22	1
C6(Carbamidomethyl)	0.0000	3.22	2	1628.68596	4.95	43.92	0
C1(Carbamidomethyl); N4(Deamidated); C5(Carbamidomethyl)	0.0000	3.16	2	1233.53801	8.20	75.94	0
	0.0000	3.15	2	931.56706	6.32	41.41	1
	0.0000	3.10	3	1190.73420	7.21	38.84	2
	0.0000	3.01	2	1019.66686	5.54	36.80	2
N1(Deamidated)	0.0000	2.98	3	1191.71912	7.97	51.32	2
	0.0000	2.87	3	1180.62137	5.93	21.17	2
	0.0000	2.55	2	891.57109	5.44	51.91	1
N4(Deamidated)	0.0000	2.51	2	892.55681	7.34	46.70	1
	0.0000	2.44	2	881.45940	4.99	32.75	1
M4(Oxidation)	0.0000	2.18	2	897.45372	4.24	29.04	1
C6(Carbamidomethyl); N12(Deamidated)	0.0000	2.10	2	1629.68389	13.48	53.49	0
	0.0000	2.03	2	778.48723	6.50	31.53	1
C1(Carbamidomethyl)	0.0000	2.03	2	1508.73113	2.27	86.84	0
