# Supplementary material for: Various Bee Pheromones Binding Affinity, Exclusive Chemosensillar Localization, and Key Amino Acid Sites Reveal the Distinctive Characteristics of Odorant-Binding Protein 11 in the Eastern Honey Bee, Apis cerana
Source: Front Physiol. 2018 Apr 23;9:422. doi: 10.3389/fphys.2018.00422 (PMC5924804; doi:10.3389/fphys.2018.00422)
Supplement: Supplementary file 6 [file Image2.PDF]

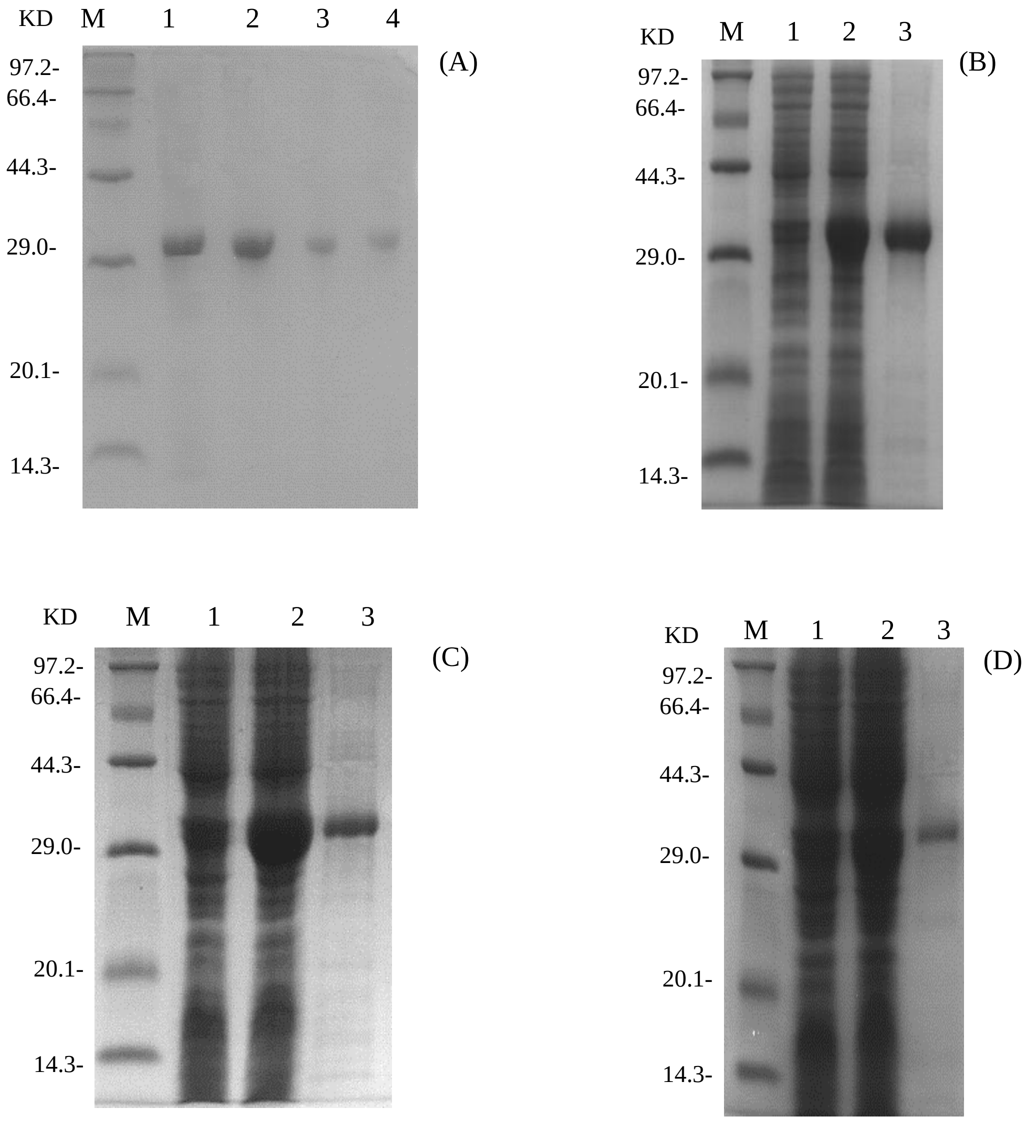

Fig.S2. Expression and purification of the three site-directed mutagenesis of AcerOBP11

(A) represents the purification of AcerOBP11 wild-type and three mutant. M is the protein molecular weight marker. Line 1 is AcerOBP11 wild-type, line 2, 3, 4 is the mutant protein of Ile140G, Phe101G, Ile97G, respectively.

(B), (C) and (D) represent the Expression and purification of the mutant protein of Ile140G, Phe101G, Ile97G, respectively. Lane M is the protein molecular weight marker. Lane 1 and 2 represent the whole of lysate including pET32a-*AcerOBP11* plasmid without and with induction of 1 mmol·L^{-1} IPTG, respectively. Lane 3 represents purified recombinant AcerOBP11 mutant proteins.
