# Supplementary material for: Various Bee Pheromones Binding Affinity, Exclusive Chemosensillar Localization, and Key Amino Acid Sites Reveal the Distinctive Characteristics of Odorant-Binding Protein 11 in the Eastern Honey Bee, Apis cerana
Source: Front Physiol. 2018 Apr 23;9:422. doi: 10.3389/fphys.2018.00422 (PMC5924804; doi:10.3389/fphys.2018.00422)
Supplement: Supplementary file 7 [file Image3.PDF]

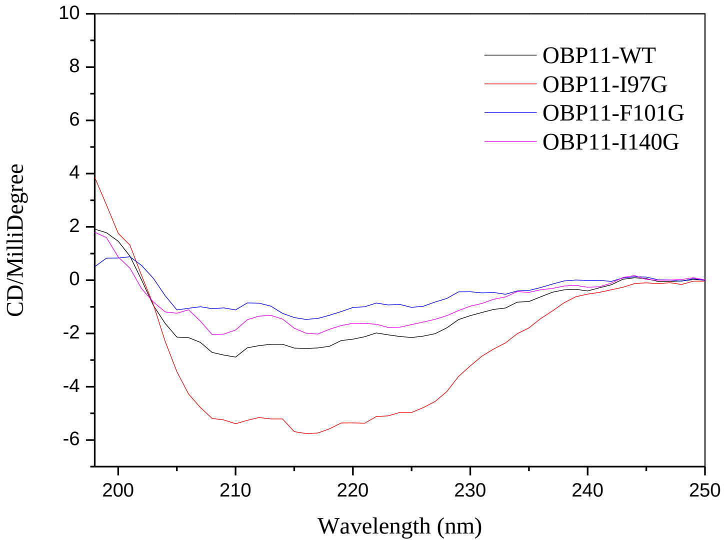

Fig.S3 Circular dichroism (CD) spectra of AcerOBP11 and three mutant proteins.

	OBP11-WT	OBP11-I97G	OBP11-F101G	OBP11-I140G
Helix	24.2	50.9	8.5	14.7
Beta	40.8	0	69.5	61.7
Turn	9.2	16.7	3.1	3
Random	25.8	32.4	18.9	20.6
Total	100	100	100	100
RMS Value	22.692	14.231	71.095	32.327
