# Supplementary material for: Various Bee Pheromones Binding Affinity, Exclusive Chemosensillar Localization, and Key Amino Acid Sites Reveal the Distinctive Characteristics of Odorant-Binding Protein 11 in the Eastern Honey Bee, Apis cerana
Source: Front Physiol. 2018 Apr 23;9:422. doi: 10.3389/fphys.2018.00422 (PMC5924804; doi:10.3389/fphys.2018.00422)
Supplement: Supplementary file 8 [file Image4.PDF]

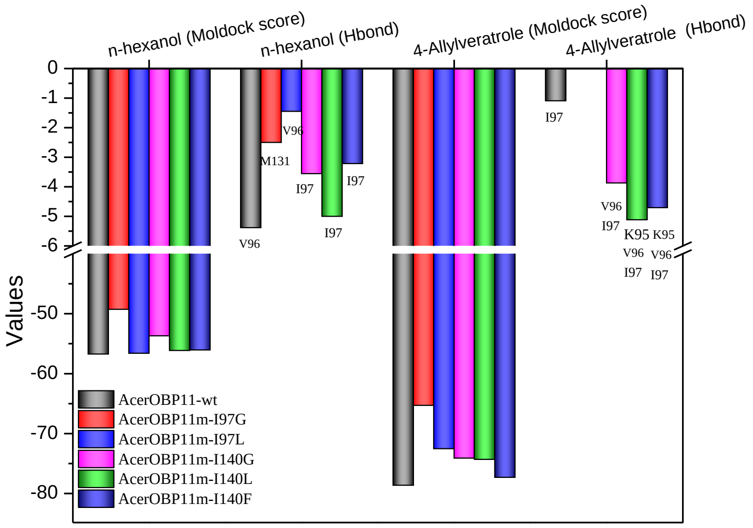

Figure S4. Predicted energies of AcerOBP11 mutants binding with ligands by the analysis of docking. It was evidently that the energies of all AcerOBP11 wild-type were always the lowest. When the Ile97/140 in AcerOBP11-wide-type were manually substituted to Leu97/140 or Phe140, respectively, the mutants of m-Ile97/140Gly were the highest, whereas the other predicted mutants of m-Ile97Leu/Ile140Leu(Phe) had slightly higher energies than AcerOBP11 wild-type.
